# Evidence of altered haemostasis in an ovine model of venovenous extracorporeal membrane oxygenation support

**DOI:** 10.1186/s13054-017-1788-9

**Published:** 2017-07-29

**Authors:** Margaret R. Passmore, Yoke L. Fung, Gabriela Simonova, Samuel R. Foley, Sara D. Diab, Kimble R. Dunster, Michelle M. Spanevello, Charles I. McDonald, John-Paul Tung, Natalie M. Pecheniuk, Karen Hay, Kiran Shekar, John F. Fraser

**Affiliations:** 1 Critical Care Research Group, University of Queensland and the Prince Charles Hospital, Brisbane, Australia; 2 School of Health and Sport Sciences, University of the Sunshine Coast, Sippy Downs, Australia; 3 Research and Development, Australian Red Cross Blood Service, Brisbane, Australia; 4 Cancer Care Services, Royal Brisbane and Women’s Hospital, Brisbane, Australia; 5 University of Queensland, Brisbane, Australia; 6 School of Biomedical Sciences, Faculty of Health, Queensland University of Technology, Brisbane, Australia; 7 QIMR Berghofer Metro North Hospital and Health Service Statistics Unit, Brisbane, Australia

**Keywords:** Extracorporeal membrane oxygenation, Thromboelastometry, Coagulation, Haemostasis, Fibrinogen, Platelet aggregation

## Abstract

**Background:**

Extracorporeal membrane oxygenation (ECMO) is a life-saving modality used in the management of cardiopulmonary failure that is refractory to conventional medical and surgical therapies. The major problems clinicians face are bleeding and clotting, which can occur simultaneously. To discern the impact of pulmonary injury and ECMO on the host’s haemostatic response, we developed an ovine model of smoke-induced acute lung injury (S-ALI) and ECMO. The aims of this study were to determine if the ECMO circuit itself altered haemostasis and if this was augmented in a host with pulmonary injury.

**Methods:**

Twenty-seven South African meat merino/Border Leicester Cross ewes underwent instrumentation. Animals received either sham injury (*n* = 12) or S-ALI (*n* = 15). Control animal groups consisted of healthy controls (ventilation only for 24 h) (*n* = 4), ECMO controls (ECMO only for 24 h) (*n* = 8) and S-ALI controls (S-ALI but no ECMO for 24 h) (*n* = 7). The test group comprised S-ALI sheep placed on ECMO (S-ALI + ECMO for 24 h) (*n* = 8). Serial blood samples were taken for rotational thromboelastometry, platelet aggregometry and routine coagulation laboratory tests. Animals were continuously monitored for haemodynamic, fluid and electrolyte balances and temperature. Pressure-controlled intermittent mandatory ventilation was used, and mean arterial pressure was augmented by protocolised use of pressors, inotropes and balanced fluid resuscitation to maintain mean arterial pressure >65 mmHg.

**Results:**

Rotational thromboelastometry, platelet aggregometry and routine coagulation laboratory tests demonstrated that S-ALI and ECMO independently induced changes to platelet function, delayed clot formation and reduced clot firmness. This effect was augmented with the combination of S-ALI and ECMO, with evidence of increased collagen-induced platelet aggregation as well as changes in factor VIII (FVIII), factor XII and fibrinogen levels.

**Conclusions:**

The introduction of an ECMO circuit itself increases collagen-induced platelet aggregation, decreases FVIII and von Willebrand factor, and induces a transient decrease in fibrinogen levels and function in the first 24 h. These changes to haemostasis are amplified when a host with a pre-existing pulmonary injury is placed on ECMO. Because patients are often on ECMO for extended periods, longer-duration studies are required to characterise ECMO-induced haemostatic changes over the long term. The utility of point-of-care tests for guiding haemostatic management during ECMO also warrants further exploration.

## Background

Extracorporeal membrane oxygenation (ECMO) is a life-saving modality used in the management of cardiopulmonary failure that is unresponsive to conventional medical and surgical therapies. Despite its benefits, survival rates for ECMO are only 84%, 67% and 66% when used for respiratory failure and 62%, 67% and 56% when used for cardiac failure in neonates, paediatric patients and adults, respectively [1]. Bleeding events remain a common and serious complication during ECMO, occurring in approximately 30% of patients [2–4]. Authors of some reports have proposed that contact between the patient’s blood and the ECMO circuit can lead to activation of the coagulation, fibrinolysis and complement pathways [5]. The shift to a pro-coagulant state appears to be mediated primarily by thrombin, whilst there is an excessive fibrinolytic tendency mediated by plasmin, with the result being consumption of clotting factors, impaired platelet function, thrombocytopenia, and fibrinolysis [5]. The goal of the clinician is to minimise bleeding and transfusion requirements whilst avoiding micro- or macro-thrombus formation in the circuit and within the patient’s cardiovascular system [6]. This tightrope is a dynamic process; hence, a more complete understanding of how haemostasis is altered may improve bleeding management strategies and thus to reduce morbidity and mortality.

Unfractionated heparin is the systemic anti-coagulant most widely used during ECMO and is monitored primarily by activated clotting time (ACT). Viscoelastic tests using rotational thromboelastometry (ROTEM®) are gaining popularity because they assess whole blood coagulation and thus provide information on the dynamics of clot development, stabilisation and dissolution. A number of small studies suggest that ROTEM®-guided coagulation management could reduce bleeding episodes in ECMO patients [7, 8]. Whole blood platelet aggregometry using the cobas Multiplate® (Roche Diagnostics, Rotkreuz, Switzerland) is also increasing in use with a recent study demonstrating decreased platelet aggregation in ECMO patients [8].

Patients who require ECMO are critically ill, thus making it difficult to discern the relative contributions of a patient’s underlying pathology from the insult of the ECMO circuit per se. We hypothesised that the ECMO circuit itself alters haemostasis and that this becomes more pronounced in the presence of a pre-existing lung injury. To discern the ECMO effect from that of the underlying illness, we used an established ovine model of lung injury and placed the animal on ECMO support. Haemostasis was monitored using conventional coagulation assays, thromboelastometry and platelet aggregometry to determine how each physiological insult alters coagulation in this scenario.

## Methods

### Extracorporeal membrane oxygenation ovine model

This study was approved by the animal research and ethics committee of the Queensland University of Technology and the University of Queensland (approval numbers 1100000053 and 1000000025) and adhered to the Australian Code for the Care and Use of Animals for Scientific Purposes, Eighth Edition, 2013 (the code), of the National Health and Medical Research Council. A total of 27 South African meat merino (SAMM)/Border Leicester Cross ewes weighing between 39 and 58 kg (mean 49.5 kg) were randomly divided into four groups. Animals received either sham injury (*n* = 12) or smoke-induced acute lung injury (S-ALI) (*n* = 15) as detailed previously [9]. Sham injury animals were divided into healthy controls (ventilation only for 24 h) (*n* = 4) and ECMO controls (ECMO only for 24 h) (*n* = 8). S-ALI animals were divided into controls (S-ALI only, no ECMO for 24 h) (*n* = 7) and the test group of S-ALI + ECMO (*n* = 8) for 24 h. ECMO was instituted as per our previously validated model [10]. Briefly, animals placed on ECMO had a 21- to 23-French femoral venous cannula (Medtronic, Minneapolis, MN, USA) inserted into the internal jugular vein and positioned in the distal inferior vena cava (access cannula). A 19- to 21-French venous cannula (Medtronic) was placed at the superior vena cava-right atrium junction (return cannula). Intra-cardiac echocardiography was used to confirm the cannula position. A Jostra ROTAFLOW centrifugal pump (Maquet Cardiopulmonary, Rastatt, Germany) was used, and target flow rates of 3–4 L/minute were maintained. Porcine mucous heparin (1000 U; Pfizer Australia, West Ryde, Australia) was added to the circuit, and a heparin infusion was commenced at 4 U/kg/h to obtain a target ACT between 220 and 300 seconds. Sheep were continuously monitored for haemodynamics, fluid and electrolyte balances, and temperature. Pressure-controlled mandatory ventilation based on previous ovine models was used [11, 12]. An arterial partial pressure of carbon dioxide of approximately 30 mmHg and arterial partial pressure of oxygen >80 mmHg were managed via ECMO gas sweep. Mean arterial pressure was augmented by protocolised use of pressors, inotropes and balanced fluid resuscitation to maintain mean arterial pressure >65 mmHg. Bolus fluids and increased maintenance fluids (up to 500 ml/h) were administered when a persistent decrement in ECMO blood flows was observed and other potential causes for the same were excluded.

### Blood sampling

Arterial blood samples and blood gas measurements (temperature, pH, ionised calcium) were collected at baseline; 15 minutes post-S-ALI (or sham injury) (S0), before ECMO commencement (S2); and 1, 2, 6, 12, 18 and 24 h post-ECMO. Blood samples were also taken for ROTEM® analysis, full blood examination (FBE) and Multiplate® whole blood platelet aggregometric testing. Samples for routine coagulation tests were centrifuged twice (15 minutes, 4 °C, 3000 × *g*) to obtain platelet-poor plasma and were subsequently stored at −80 °C until further analysis.

### Full blood examination and routine coagulation tests

An FBE using the veterinary mode of the AcT diff™ haematology analyser (Beckman Coulter Australia Pty Ltd, Lane Cove West, Australia) was performed to assess the white cell count (WCC), red cell count (RCC), haemoglobin (Hb), haematocrit (Hct) and platelet count. Prothrombin time (PT), activated partial thromboplastin time (aPTT), factor VIII (FVIII) and FXII, anti-thrombin (AT), fibrinogen using the Clauss method, protein C, von Willebrand factor antigen (vWAg), thrombin clotting time (TCT) and d-dimer were performed on the ACL TOP® analyser (Instrumentation Laboratory/Werfen Australia, Artarmon, Australia) or the Stago STA-R Evolution analyser (Diagnostica Stago, Doncaster, Australia) following the manufacturer’s instructions. ACT was measured in fresh whole blood using kaolin tubes with the HEMOCHRON® 401 coagulation analyser (Soma Technology, Bloomfield, CT, USA).

### Whole blood platelet function (Multiplate®)

Whole blood platelet aggregometry was measured by change in impedance and expressed in aggregation units using the Multiplate® 5.0 platelet function analyser. Hirudin-anticoagulated blood was tested using adenosine diphosphate (ADP; 6.4 μmol/L) and collagen (3.2 μg/ml) agonists [13, 14].

### ROTEM®

Whole blood clot formation profiles were recorded by ROTEM® (Haemoview Diagnostics, Brisbane, Australia) with the EXTEM (thromboplastin-initiated coagulation), INTEM (contact factor-initiated coagulation), FIBTEM (thromboplastin-initiated coagulation with the platelet inhibitor cytochalasin D) and HEPTEM (contact factor-initiated coagulation with heparinase) activating reagents in accordance with the manufacturer’s instructions. Parameters evaluated included clotting time (CT), clot formation time (CFT) and maximum clot firmness (MCF).

### Statistical analysis

To determine the effect of the experimental group on each outcome, a mixed effects linear regression model was fitted with random effects for sheep and time (within sheep). Potential confounders included the laboratory where routine and specialised coagulation testing was performed, weight of the animal at baseline, fluid balance and the ratio of INTEM-CT to HEPTEM-CT (used as a proxy measure to adjust for potential confounding due to heparin dosage). Eligible confounders with univariable *P* values <0.2 were entered into a multivariable model, and a stepwise backwards elimination process was applied until all variables remained significant at the 0.05 level. Statistical analyses were performed using the STATA^TM^ 13 statistical software package (StataCorp, College Station, TX, USA).

## Results

### Physiological, haematological and routine coagulation parameters

**Fig. 1:**
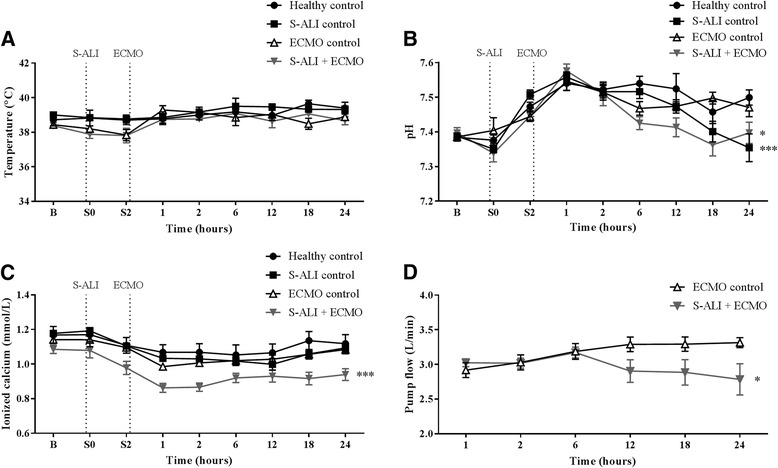
The effect of ECMO on physiological parameters. Temperature remained constant **a**, while S-ALI controls and S-ALI + ECMO animals had a decreased pH **b**. Ionised calcium levels **c** and the circuit flow rate **d** were decreased in S-ALI + ECMO compared with healthy controls. Data are presented as mean ± SEM. *P < 0.05, ***P < 0.001 versus healthy controls. *n* = 4 healthy controls, *n* = 8 ECMO controls, *n* = 7 S-ALI controls, *n* = 8 S-ALI + ECMO. *ECMO* Extracorporeal membrane oxygenation, *S-ALI* Smoke-induced acute lung injury, *B* Baseline

Sheep body temperature remained constant throughout the experiment (Fig. 1a). Significant decreases in pH (Fig. 1b) were seen in the S-ALI control (*P* < 0.001) and S-ALI + ECMO groups (*P* < 0.05) compared with healthy controls, although this was still within the normal range for ovine pH (7.32–7.5). S-ALI + ECMO groups also had decreased ionised calcium levels (*P* < 0.001) (Fig. 1c) and a significantly decreased circuit flow rate (*P* < 0.05) (Fig. 1d) compared with healthy controls. The mean and SD of all haematological and plasma-based coagulation parameters at selected time points are detailed in Table 1. Baseline measurements for FBE as well as routine and specialised coagulation tests did not differ significantly across the experimental groups. Results from each group were compared with those of the healthy control group. WCC (*P* = 0.004), RCC (*P* = 0.01) and Hb (*P* < 0.001) increased in the S-ALI controls. In contrast, WCC (*P* = 0.01), RCC (*P* < 0.001) and Hb (*P* < 0.001) levels were lower in the ECMO controls. S-ALI + ECMO animals also had lower WCC (*P* < 0.001). Platelet count remained unchanged across all groups. A prolonged PT was observed in both S-ALI controls (*P* < 0.001) and S-ALI + ECMO (*P* < 0.001) groups after 12 h of ECMO. aPTT, TCT and d-dimer did not differ significantly in any groups compared with healthy controls. Fibrinogen, FVIII and vWAg levels were lower in ECMO controls (*P* < 0.001). Fibrinogen, AT, FXII and protein C levels were lower in S-ALI controls (*P* < 0.001). With the combination of S-ALI + ECMO, there were reductions in coagulation factors FVIII and FXII as well as fibrinogen and vWAg. The levels of naturally occurring anti-coagulants AT and protein C were also lower in this group (*P* < 0.001). Predicted marginal means with 95% CIs over experimental group for measures of interest are shown in Table 2 along with overall Wald *P* values derived from mixed effects regression models.

**Table 1:** Routine and specialised haemostatic parameters by experimental group at selected time points

	Ventilation only		S-ALI control		ECMO control		S-ALI + ECMO	
	B	24 h	B	24 h	B	24 h	B	24 h
Full blood examination								
WCC, ×10^9^/L	9.8 (3.1)	10.2 (2.2)	7.8 (2.1)	21.8 (8.3)^a^	7.0 (2.2)	7.9 (1.7)^a^	6.6 (1.2)	9.1 (3.8)^a^
RCC, ×10^12^/L	8.7 (0.68)	6.2 (0.6)	8.5 (0.78)	8.9 (1.36)^a^	8.0 (1.2)	4.8 (0.54)^a^	8.3 (0.87)	7.3 (1.2)
Hb, g/L	97.8 (9.1)	70.3 (6.1)	96.1 (9.4)	99.1 (12)^a^	89.3 (12)	54.3 (5.4)^a^	93.4 (11)	83.5 (12)
Hct	0.28 (0.02)	0.20 (0.02)	0.28 (0.03)	0.29 (0.04)^a^	0.26 (0.04)	0.16 (0.01)^a^	0.28 (0.03)	0.24 (0.04)
PLT, ×10^9^/L	341 (173)	190 (110)	355 (186)	203 (111)	471 (95)	260 (80)	364 (140)	199 (60)
Routine coagulation tests								
PT, seconds	14.5 (1)	17.3 (1)	15.9 (1.1)	34.4 (5.4)^a^	12.9 (0.6)	17.4 (2.1)	14.3 (0.9)	35 (6.7)^a^
aPTT, seconds	33 (6)	139 (76)	31 (6)	165 (42)	26 (4.6)	122 (64)	30 (6)	158 (57)
Fibrinogen, g/L	2.5 (0.54)	3.5 (0.13)	2.1 (0.62)	2.5 (0.34)^a^	2.9 (0.47)	2.6 (0.16)^a^	3.0 (0.97)	1.5 (0.5)^a^
d-dimer, ng/ml	215 (85)	198 (70)	146 (44)	139 (82)	435 (192)	343 (217)	406 (213)	213 (136)
TCT, seconds	13 (0)	21 (7)	13 (0)	81 (84)	12 (1)	156 (81)	13 (1)	44 (69)
Specialised coagulation tests								
FVIII, %	1531 (413)	1390 (414)	1347 (417)	1544 (238)	891(235)	408 (51)^a^	904 (166)	531 (242)^a^
FXII, %	103 (17.7)	75 (10)	96 (30.9)	29 (11.5)^a^	151 (37.7)	67 (12.4)	107 (20.2)	16 (6.7)^a^
Protein C, %	21.5 (2.5)	21.5 (1.3)	21 (7.4)	12.1 (3.2)	55 (13.4)	50 (11.2)	46 (11.4)	14 (4.5)^a^
vWAg, %	136 (22.3)	130 (25.8)	96 (31.9)	140 (18.2)	94 (47.6)	88 (10.9)^a^	129 (30)	96 (25.3)^a^
AT, %	85.8 (7.3)	58.3 (6.6)	84.1 (3.9)	27.1 (3.2)^a^	93.7 (8.8)	62.7 (6)	92.3 (1.9)	22 (4.3)^a^

*Abbreviations: aPTT* Activated partial thromboplastin time, *AT* Anti-thrombin, *B* baseline, *ECMO* Extracorporeal membrane oxygenation, *FVIII* Factor VIII, *FXII* Factor XII, *Hb* Haemoglobin, *Hct* Haematocrit, *PLT* Platelets, *PT* Prothrombin time, *RCC* Red cell count, *S-ALI* Smoke-induced acute lung injury, *TCT* Thrombin clotting time, *vWAg* von Willebrand factor antigen, *WCC* White cell count

Data are presented as mean (±SD)

^a^ *P* < 0.05 over time vs ventilation only

**Table 2:** Predicted marginal mean values and 95% CIs for haemostatic parameters, by experimental group and overall Wald *P* values derived from mixed effects regression models

	Ventilation only	S-ALI control	ECMO control	S-ALI + ECMO	Wald *P* value
WCC	8.4 (6.8–9.9)	5.5 (4.4–6.7)	6.0 (4.8–7.2)	4.9 (3.8–6)	0.004
RCC	6.9 (6.3–7.5)	7.3 (6.9–7.8)	5.0 (4.5–5.5)	6.2 (5.7–6.6)	<0.001
Hb	75 (70–81)	87 (83–91)	55 (50–60)	73 (69–77)	<0.001
PLT	195 (121–269)	248 (188–309)	238 (185–290)	207 (155–260)	0.609
PT	16 (13.4–18.6)	20 (17.7–22.2)	18.9 (16.7–21)	23.7 (22.1–25.3)	0.007
aPTT	113 (89–137)	117 (96–139)	100 (80–119)	124 (106–141)	0.164
TCT	68 (36–99)	96 (69–122)	159 (133–184)	104 (82–127)	0.063
d-dimer	211 (98–324)	157 (71–242)	332 (232–432)	276 (195–358)	0.852
Fib(C)	2.7 (2.4–3)	2.0 (1.7–2.3)	2.2 (1.9–2.4)	1.4 (1.2–1.6)	<0.001
FVIII	1161 (1016–1307)	1082 (956–1208)	529 (410–647)	505 (415–595)	<0.001
FXII	70 (59–81)	22 (13–31)	63 (55–72)	13 (6–20)	<0.001
vWAg	135 (120–151)	130 (118–142)	92 (79–106)	103 (92–114)	<0.001
Protein C	23 (17–29)	15 (10–20)	51 (47–56)	14 (9–18)	<0.001
INTEM-CFT	323 (154–492)	332 (179–486)	211 (115–306)	235 (121–348)	0.468
INTEM-MCF	67 (55–79)	52 (41–63)	64 (57–71)	58 (52–64)	<0.001
HEPTEM-CFT	92 (35–149)	89 (46–132)	136 (85–187)	105 (65–146)	0.5416
HEPTEM-MCF	69 (65–74)	67 (63–71)	70 (65–74)	60 (57–63)	0.001
EXTEM-CFT	108 (87–130)	144 (127–160)	105 (90–121)	179 (164–194)	<0.001
EXTEM-MCF	75 (71–78)	71 (68–74)	71 (68–74)	63 (61–65)	<0.001
FIBTEM-MCF	29 (26–33)	23 (20–26)	23 (20–26)	16 (14–19)	<0.001

*Abbreviations: aPTT* Activated partial thromboplastin time, *AT* Anti-thrombin, *CFT* Clot formation time, *ECMO* Extracorporeal membrane oxygenation, *Fib(C)* Clauss fibrinogen, *FVIII* Factor VIII, *FXII* Factor XII, *Hb* Haemoglobin, *MCF* Maximum clot firmness, *PLT* Platelets, *PT* Prothrombin time, *RCC* Red cell count, *S-ALI* Smoke-induced acute lung injury, *TCT* Thrombin clotting time, *vWAg* von Willebrand factor antigen, *WCC* White cell count

Data are presented as predicted mean (95% CI)

### Platelet function (Multiplate®)

**Fig. 2:**
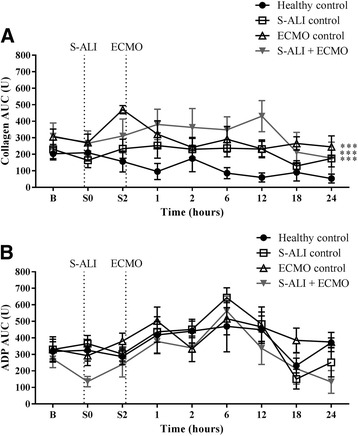
Platelet aggregometric function for each group of animals. **a** Collagen-induced AUC was significantly higher in S-ALI controls, ECMO controls and S-ALI + ECMO groups, while **b** ADP was not significantly different. Data are presented as mean ± SEM. ***P < 0.001 versus healthy controls. *n* = 4 healthy controls, *n* = 8 ECMO control, *n* = 7 S-ALI controls, *n* = 8 S-ALI + ECMO. *ADP* Adenosine diphosphate; *B* Baseline, *ECMO* Extracorporeal membrane oxygenation, *S-ALI* Smoke-induced acute lung injury

Relative to healthy controls, collagen-induced platelet function was significantly higher in S-ALI controls (*P* = 0.001), ECMO controls (*P* < 0.001) and S-ALI + ECMO (*P* < 0.001) experimental groups (Fig. 2a). There was no significant difference in ADP platelet aggregation across any of the experimental groups (Fig. 2b).

### Thromboelastometry

**Fig. 3:**
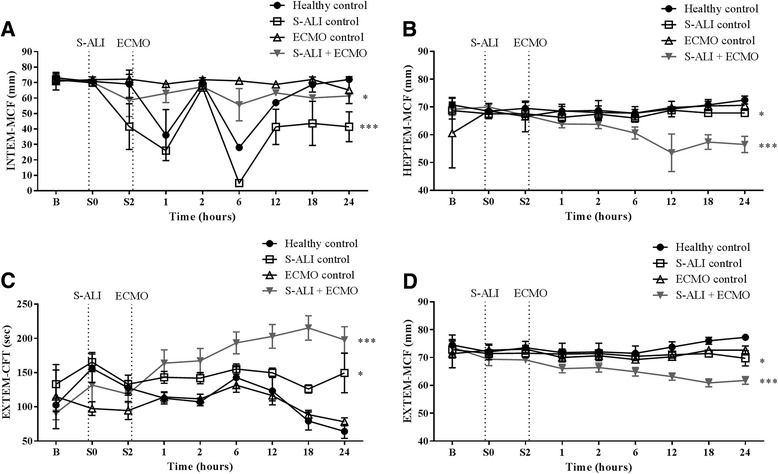
The effect of ECMO on INTEM, HEPTEM and EXTEM parameters. INTEM and HEPTEM-MCF **a** and **b** in the S-ALI controls and S-ALI + ECMO groups was significantly lower than in healthy controls. **c** EXTEM-CFT was prolonged in both S-ALI controls and S-ALI + ECMO. **d** EXTEM-MCF was subsequently lower in both S-ALI controls and S-ALI + ECMO. Data are presented as mean ± SEM. *P < 0.05, ***P < 0.001 versus healthy controls. *n* = 4 healthy controls, *n* = 8 ECMO controls, *n* = 7 S-ALI controls, *n* = 8 S-ALI + ECMO. *B* Baseline, *CFT* Clot formation time, *ECMO* Extracorporeal membrane oxygenation, *MCF* Maximum clot firmness, *S-ALI* Smoke-induced acute lung injury

S-ALI controls (*P* < 0.001) and the S-ALI + ECMO group (*P* = 0.03) had a significantly lower INTEM-MCF (Fig. 3a) than healthy controls. Both S-ALI controls (*P* = 0.007) and S-ALI + ECMO (*P* < 0.001) animals had a significantly lower HEPTEM-MCF (Fig. 3b). There was no significant difference in INTEM or HEPTEM-CFT across any of the experimental groups (data not shown). EXTEM-CFT was extended in S-ALI controls (*P* = 0.011) and further protracted in S-ALI + ECMO (*P* < 0.001) experimental animals (Fig. 3c). In addition, the overall clot quality as measured by the EXTEM-MCF was also lower in S-ALI controls (*P* = 0.031) and further decreased in S-ALI + ECMO (*P* < 0.001) (Fig. 3d).

**Fig. 4:**
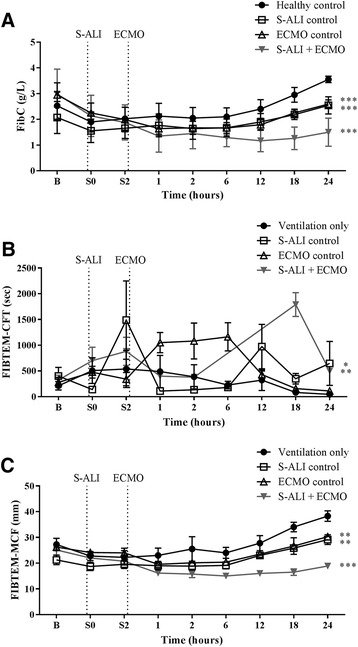
The effect of ECMO on FIBTEM parameters. **a** Clauss fibrinogen was significantly decreased in all three groups compared with healthy controls. **b** FIBTEM-CFT was prolonged in both S-ALI controls and S-ALI + ECMO groups, while **c** FIBTEM-MCF was lower in S-ALI controls, ECMO controls and S-ALI + ECMO. Data are presented as mean ± SEM. *P < 0.05, **P < 0.01, ***P < 0.001 versus healthy controls. *n* = 4 healthy controls, *n* = 8 ECMO controls, *n* = 7 S-ALI controls, *n* = 8 S-ALI + ECMO. *B* Baseline, *CFT* Clot formation time, *ECMO* Extracorporeal membrane oxygenation, *FibC* Clauss fibrinogen, *MCF* Maximum clot firmness, *S-ALI* Smoke-induced acute lung injury

Fibrinogen levels in all three experimental groups (*P* < 0.001) were significantly lower than in healthy controls (Fig. 4a). FIBTEM-CFT was prolonged in both S-ALI controls (*P* = 0.016) and the S-ALI + ECMO group (*P* = 0.001) (Fig. 4b). FIBTEM-MCF was lower in S-ALI controls (*P* < 0.001) and ECMO controls (*P* = 0.001) and was further reduced in S-ALI + ECMO (*P* < 0.001) groups (Fig. 4c).

## Discussion

In clinical practice, it is the critically ill who are placed on ECMO. This ovine smoke injury and ECMO model has revealed that the introduction of an ECMO circuit alone leads to an increase in collagen-induced platelet aggregation and a reduction in the levels of coagulation factors FVIII, FXII and fibrinogen. When a host with S-ALI is placed on ECMO, these haemostatic alterations are augmented.

Studies on the impact of ECMO on platelet number document variable results. A number of case series and reviews suggest the ECMO circuit may lead to thrombocytopenia through platelet consumption [15–17]; however, two recent studies demonstrated that ECMO is not associated with decreased platelet numbers [18, 19]. Consistent with these latter reports, we observed no significant decrease in platelet numbers in our ovine ECMO model.

While platelet numbers were unaffected by ECMO, it is possible that haemostasis in our ovine ECMO model may have been impacted by perturbations in platelet function. To investigate this possibility, we conducted whole blood platelet function assays. We and others have previously demonstrated the utility of human platelet function assays in the ovine setting [13, 14]. The platelet ADP response in SAMM/Border Leicester Cross sheep is comparable to that of humans [14], and in our study, there was no change with ADP-induced platelet aggregation in any of the sheep groups compared with healthy controls. In contrast, researchers in several studies have reported an impaired platelet response with ADP in adults [8, 20] as well as children on ECMO [21]. Patients are often on ECMO for extended time periods, and because our study involved only a 24-h observation period, it is conceivable that ADP-dependent platelet dysfunction may develop with prolonged exposure. While the collagen response in SAMM/Border Leicester Cross sheep is lower than that of humans [14], we observed significant increases in collagen-triggered platelet function with S-ALI and ECMO individually and sequentially. The shear stress from the ECMO circuit is thought to damage endothelial cells, thus exposing collagen and inducing platelet aggregation [22]. In addition, shear stress has been shown to induce amplification of platelet micro-particle production and shedding [23], as well as to prime platelets for faster activation [24].

SAMM/Border Leicester Cross sheep have similar levels of von Willebrand factor (vWF) to humans, but they have significantly higher levels of FVIII [14]. Results from this study demonstrate that while S-ALI does not affect circulating FVIII or vWF, the introduction of the ECMO circuit in both ECMO controls and S-ALI + ECMO resulted in a significant reduction in vWF and FVIII levels. Because our analysis was corrected for fluid administration, this effect cannot be attributed to haemodilution alone. Rather, exposure of blood to the increased shear stress of the ECMO circuit may be causative. Increased shear stress, as seen in ventricular assist devices (VADs), total artificial hearts and aortic valve stenosis, has been reported to cause alterations in the configuration of vWF and reduced levels of high-molecular-weight multimers (HMWM) of vWF [25]. Patients on long-term VADs have been reported to develop acquired von Willebrand syndrome, which has been linked to the loss of HMWM [26, 27]. In a study of eight ECMO patients, researchers reported relatively stable FVIII levels but a decreased ristocetin cofactor to vWF ratio, indicating the possible loss of HMWM of vWF [17, 28]. While no change to FVIII levels has been recorded in human studies, we speculate that the higher levels of FVIII in sheep enabled us to detect this change more easily.

Because ROTEM is routinely used to provide rapid results for managing bleeding in our intensive care unit [29], we also applied it to assess whole blood coagulation in this model. The shortened INTEM CTs in both the ECMO groups are consistent with reduced FVIII and vWF. As expected with heparin administration, prolongation of the aPTT did not change significantly across groups. With respect to measures of the extrinsic pathway, both S-ALI controls and S-ALI + ECMO samples demonstrated an increase in PT to more than double the baseline time after 24 h of ECMO support. These results suggest that whole blood coagulation assays (thromboelastometry) may be more informative and sensitive than plasma-based assays (aPTT and PT) when monitoring ECMO patients [30].

Our results showed that both S-ALI and ECMO individually compromised fibrinogen function, and the combined insult of S-ALI + ECMO produced a cumulative effect. Interestingly, serial monitoring revealed a transient early decrease in fibrinogen level and function following the initiation of ECMO, which begins normalising after 12 h. Fibrinogen is one of the first plasma proteins to be adsorbed onto the ECMO circuit and has been shown to be associated with platelet consumption [31]. Whether fibrinogen levels return to baseline levels and function after 24 h remains to be investigated.

d-Dimer levels did not change significantly in any of the groups, but protein C and AT levels decreased with time in both S-ALI controls and S-ALI + ECMO. Studies in patients with burn injuries and inhalational trauma showed increased thrombin-AT complexes and fibrin degradation products with decreased levels of protein C and AT consistent with pulmonary coagulopathy [32]. A series of ovine models of smoke inhalation injury have shown that aerosolised anticoagulants such as heparin, recombinant human AT and recombinant activated protein C reduce airway obstruction and improve oxygenation [33–35]. Because this was a 24-h model, we were unable to determine if the compromise to the extrinsic pathway continued, plateaued or recovered with time.

As with any animal model, there were species-associated limitations. While SAMM/Border Leicester Cross sheep have aPTT, PT, TCT and fibrinogen levels similar to those in humans, they have significantly higher FVIII and half the protein C levels. In addition, coagulation measurements were performed using assays optimised for humans and were referenced against human calibration plasma with assigned values traceable to National Institute for Biological Standards and Control standards. The 24-h duration of the experiment was also a limitation because most patients remain on ECMO support for days to weeks. The complexity of the study and the high cost of the experiment prevented us from extending the duration of ECMO. Longer-duration studies are required to determine if the observed haemostatic changes are transient or stable or whether they will escalate.

The predominant challenge for a clinician caring for a patient on ECMO is making an informed assessment of the bleeding and clotting risks of each patient. Assessment of the patient’s haemostasis includes consideration of the pathophysiology, degree of organ failures and extent of tissue trauma during cannulation. Thus, a holistic approach to haemostatic management is needed to balance all these factors. This incremental ovine model enabled us to show that S-ALI + ECMO induced decreases in FVIII, FXII and vWAg and altered platelet response. While factor assays cannot be performed quickly, both ROTEM and whole blood platelet aggregometry provide rapid information on whole blood coagulation and thus may be used to guide the use of blood product support, factor replacement, anti-coagulation therapy and anti-fibrinolytics. Future research on the utility of ROTEM and whole blood platelet aggregometry of ECMO patients is required to determine their efficacy to support real-time haemostatic management in this cohort.

## Conclusions

This animal model enabled us to demonstrate that the introduction of an ECMO circuit itself alters haemostasis, specifically by increasing collagen-induced platelet aggregation and reducing levels of fibrinogen, FVIII and vWF. In addition, the model demonstrated that these haemostatic perturbations are amplified in a host with pre-existing pulmonary injury. Thus, the introduction of ECMO support on its own alters haemostasis in the first 24 h. Longer-duration studies are required to characterise the haemostatic changes beyond this time. Additional efficacy studies are also required to assess the potential of point-of-care coagulation and platelet function tests for guiding haemostatic management during ECMO.

## Abbreviations

ACT: Activated clotting time
ADP: Adenosine diphosphate
aPTT: Activated partial thromboplastin time
AT: Anti-thrombin
B: Baseline
CFT: Clot formation time
CT: Clotting time
ECMO: Extracorporeal membrane oxygenation
FBE: Full blood examination
Fib(C): Clauss fibrinogen
FVIII: Factor VIII
FXII: Factor XII
Hb: Haemoglobin
Hct: Haematocrit
HMWM: High-molecular-weight multimers
MCF: Maximum clot firmness
PLT: Platelets
PT: Prothrombin time
RCC: Red cell count
ROTEM: Rotational thromboelastometry
S-ALI: Smoke-induced acute lung injury
SAMM: South African meat merino
TCT: Thrombin clotting time
VAD: Ventricular assist device
vWAg: von Willebrand factor antigen
vWF: Von Willebrand factor
WCC: White cell count
